# Supplementary material for: Dual-barcoded shotgun expression library sequencing for high-throughput characterization of functional traits in bacteria
Source: Nat Commun. 2019 Jan 18;10:308. doi: 10.1038/s41467-018-08177-8 (PMC6338753; doi:10.1038/s41467-018-08177-8)
Supplement: Supplementary file 1 — Supplementary Information [file 41467_2018_8177_MOESM1_ESM.pdf]

SUPPLEMENTARY INFORMATION

Dual-barcoded shotgun expression library sequencing for high-throughput characterization of functional traits in bacteria

Mutalik et al.

Link to website with supplementary information:
http://genomics.lbl.gov/supplemental/dubseq18/

Supplementary Note 1:

Ridge, Lasso, and Elastic Net

The Ridge, Lasso, and Elastic Net regressions were implemented using the scikit-learn python library for machine learning. The regression was done on sparse representation of matrix A, without calculation of intercept since fragment scores were normalized (to set the median to zero). The regularization parameters were estimated using 3-fold cross validation (RidgeCV, LassoCV, and ElasticNetCV classes from the sklearn.linear_model package). The parameters were first estimated for each of 155 experiments, and then the parameters that deliver the highest R-square across all samples were selected as optimal.

The objective functions to be minimized and optimal regularization parameters for Ridge, Lasso, and Elastic Net are described below.

Ridge

Ridge is L_2 regularization with objective function:

$$||Ag - f||_2^2 + \alpha||g||_2^2$$

where $\propto$ controls the amount of regularization (shrinkage). The optimal α =1.0

Lasso

Lasso is L_1 regularization with objective function:

$$||Ag - f||_2^2 + \alpha||g||_1$$

where α controls the amount of regularization (shrinkage) and variable selection. The optimal α =3.4

Elastic Net

Elastic Net is regularization with linear combination of L_1 and L_2 terms and objective function:

$$||Ag - f||_2^2 + \alpha\,\gamma||g||_1 + \frac{\alpha(1-\gamma)}{2}||g||_2^2$$

where α controls the amount of regularization and γ defines the relative contribution of L_1 and L_2 terms/ The optimal parameters: α =3.6; γ =0.7

The regression analysis was run using optimal parameters and then manual inspection of regression results obtained from all three methods (Ridge, Elastic Net and LASSO) was performed for known gene-function associations. We observed that Ridge and Elastic Net with optimal parameters tends to significantly underestimate the fitness scores for causative genes that expected to have high positive or negative fitness scores. This underestimation is caused by shrinkage effect introduced by both regularization approaches. At the same time, the LASSO, when used with optimal parameters, seems to lack this problem and produces the most accurate scores across all three approaches. As an example, this is shown for *rcnA* gene (condition: 1.2 mM Nickel) scores calculated from Ridge, Elastic Net and LASSO approaches (**Supplementary Figure 7a**). However, LASSO with optimal parameters still did not solve OLS over fitting problem completely, and still gave the unrealistic extreme positive and extreme negative scores for neighboring genes (for example, comparison of *rcnB* and *yehA*, condition: 1mM Cobalt, **Supplementary Figure 7bc**). In comparison, NNLS had no regularization parameters, and we did not observe over fitting issues.

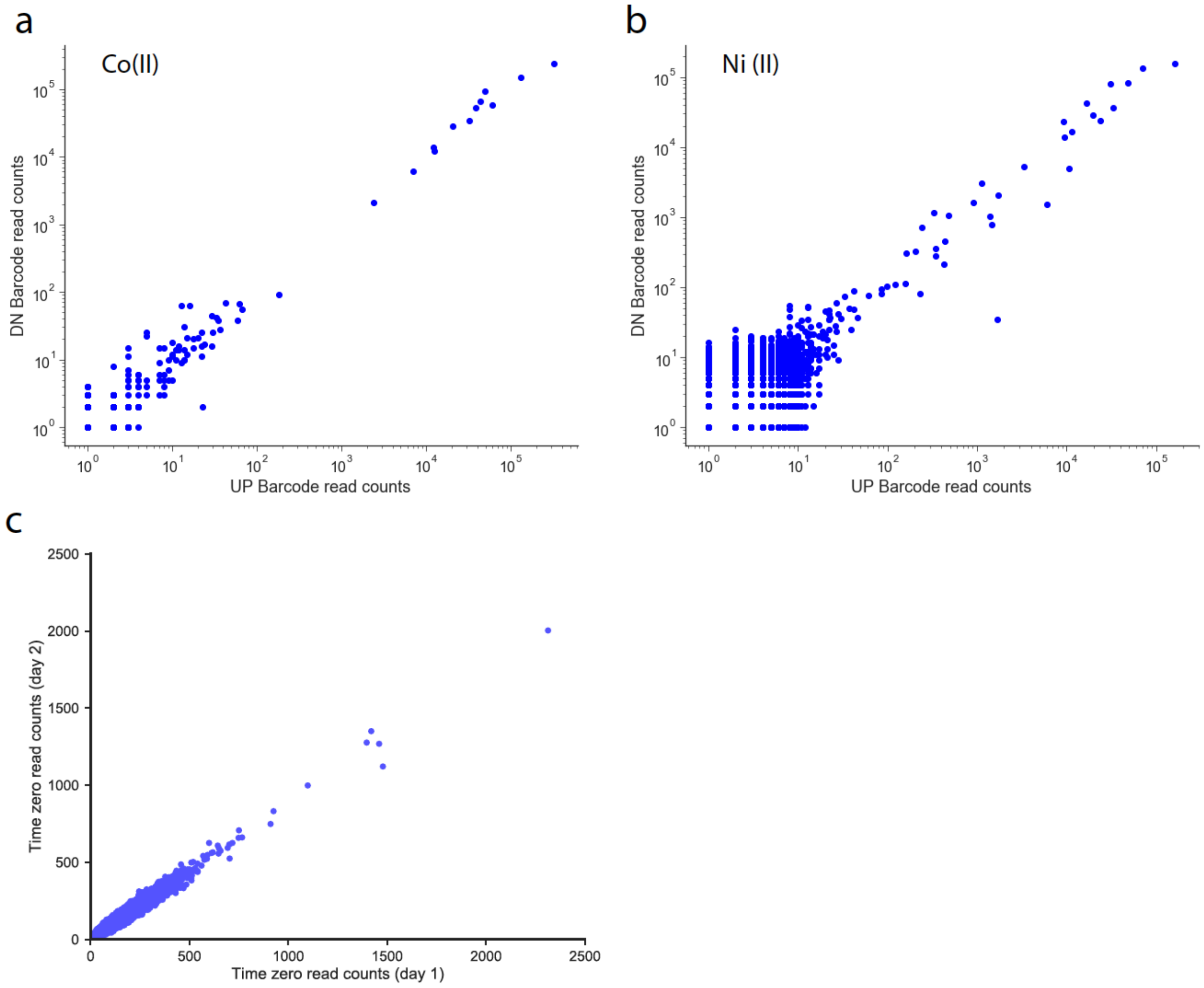

Supplementary Figure 1. BarSeq reproducibility: Comparison of UP and DOWN barcode BarSeq reads for (a) Cobalt and (b) Nickel condition. (c) Comparison of UP barcode reads for two independent start (time-zero) samples. Source data are provided as a Source Data file.

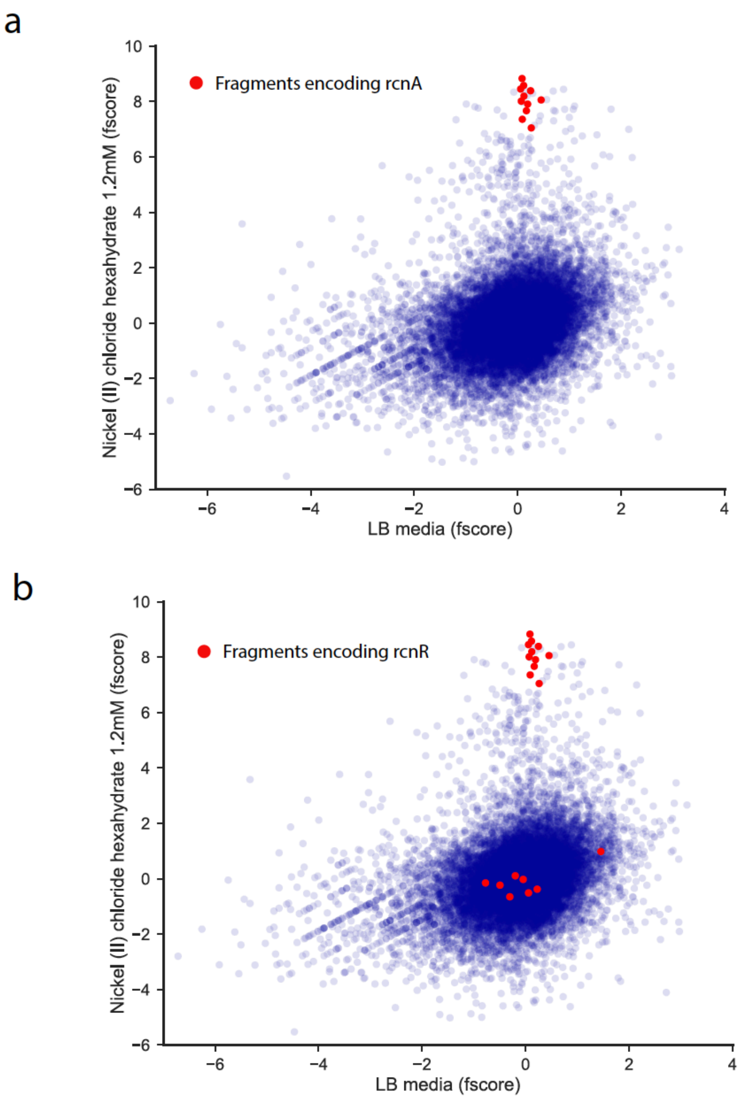

Supplementary Figure 2. Fragment score comparisons: Fragment score (fscore) comparisons for all fragments in LB (x-axis) and LB with nickel (y-axis). (a) Fragments fully covering *rcnA* are highlighted in red. (b) Fragments fully covering *rcnR* are highlighted in red. Source data are provided as a Source Data file.

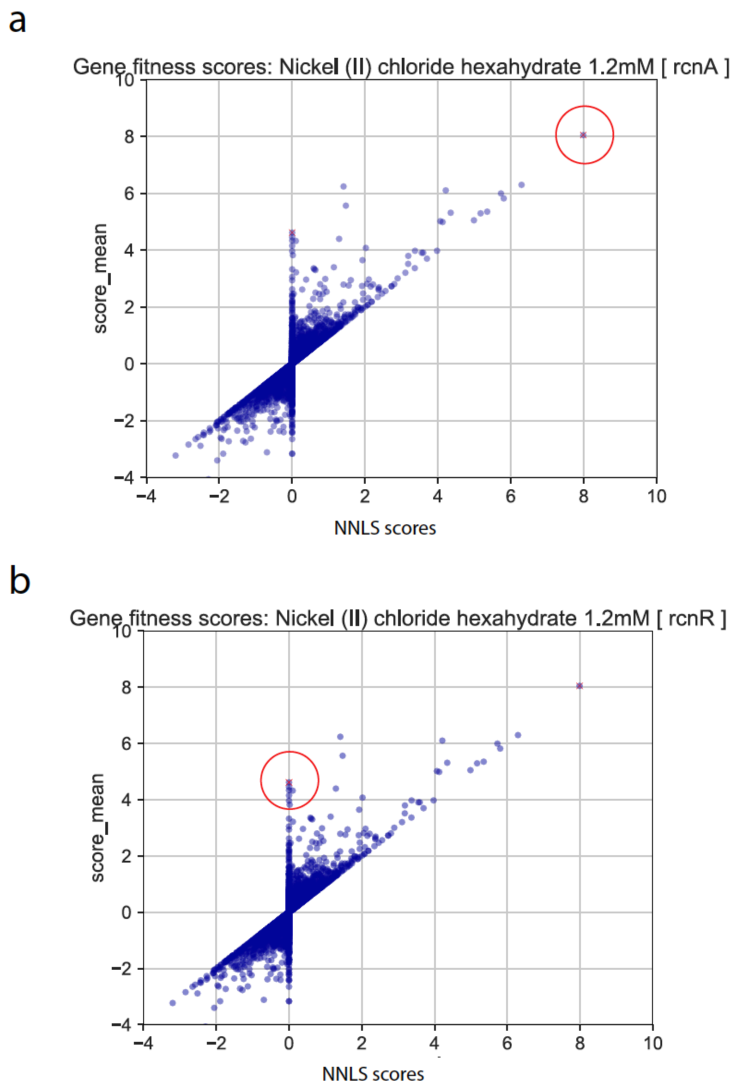

Supplementary Figure 3. Comparison of gene scores from regression analysis and mean gene scores: Comparison between gene fitness scores calculated using Non-Negative Least Squares regression (NNLS) method and the mean score method under nickel stress (a) Fitness score for *rcnA* (red circle) (b) Fitness score for *rcnR* (red circle). Source data are provided as a Source Data file.

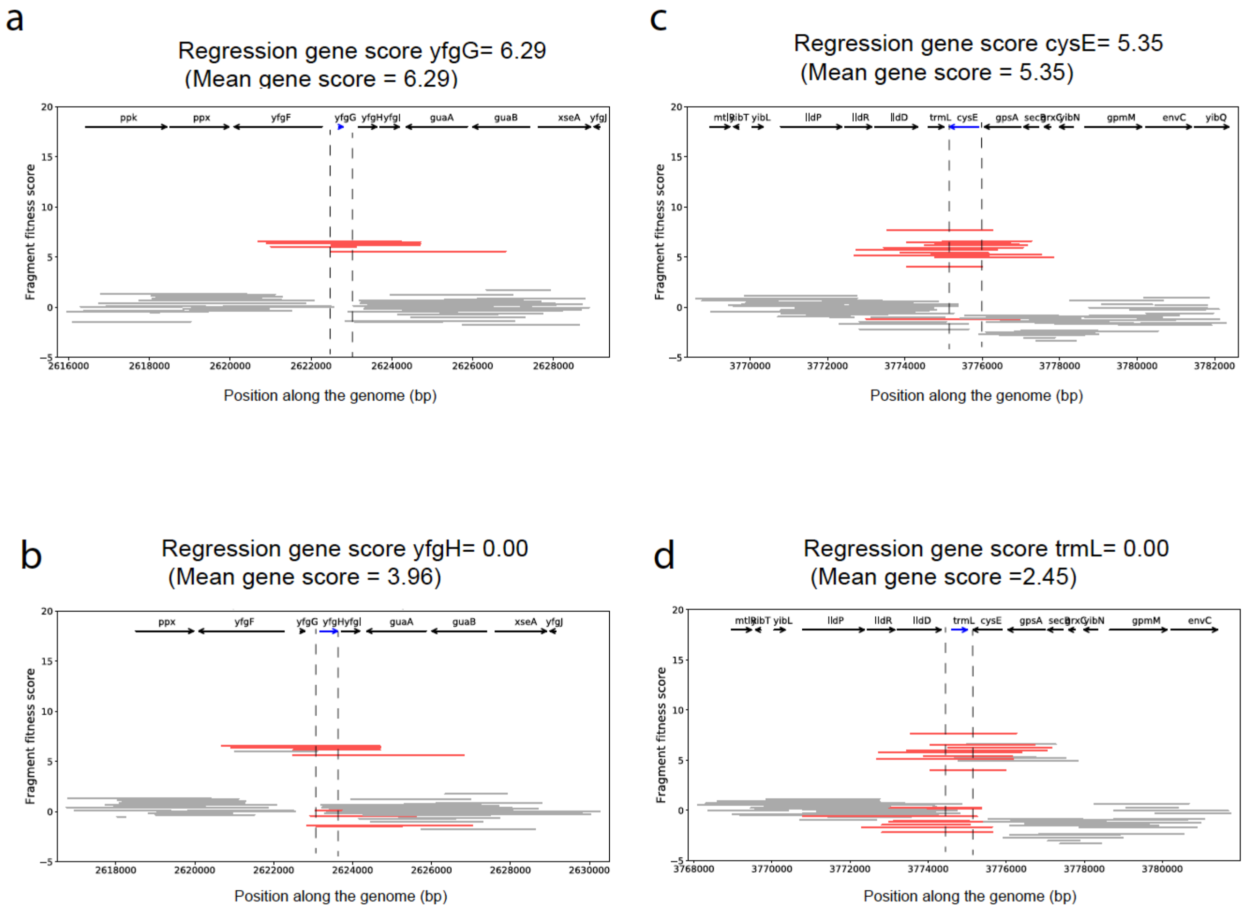

Supplementary Figure 4. Fragment and gene Dub-seq scores: Dub-seq fragment (strain) data for different regions under elevated nickel stress (y-axis). Each line shows a Dub-seq fragment with those that completely cover the indicated gene are in red. The mean and regression scores for each indicated gene are shown below each plot. Compare scores for (a) *yfgG* with (b) *yfgH,* and (c) *cysE* with (d) *trmL.* Note that the mean and regression scores for *yfgH* and *trmL* are different. The mean score is incorrectly high for *yfgH* and *trmL* and is due to the presence of *yfgG and cysE* on a number of fragments.

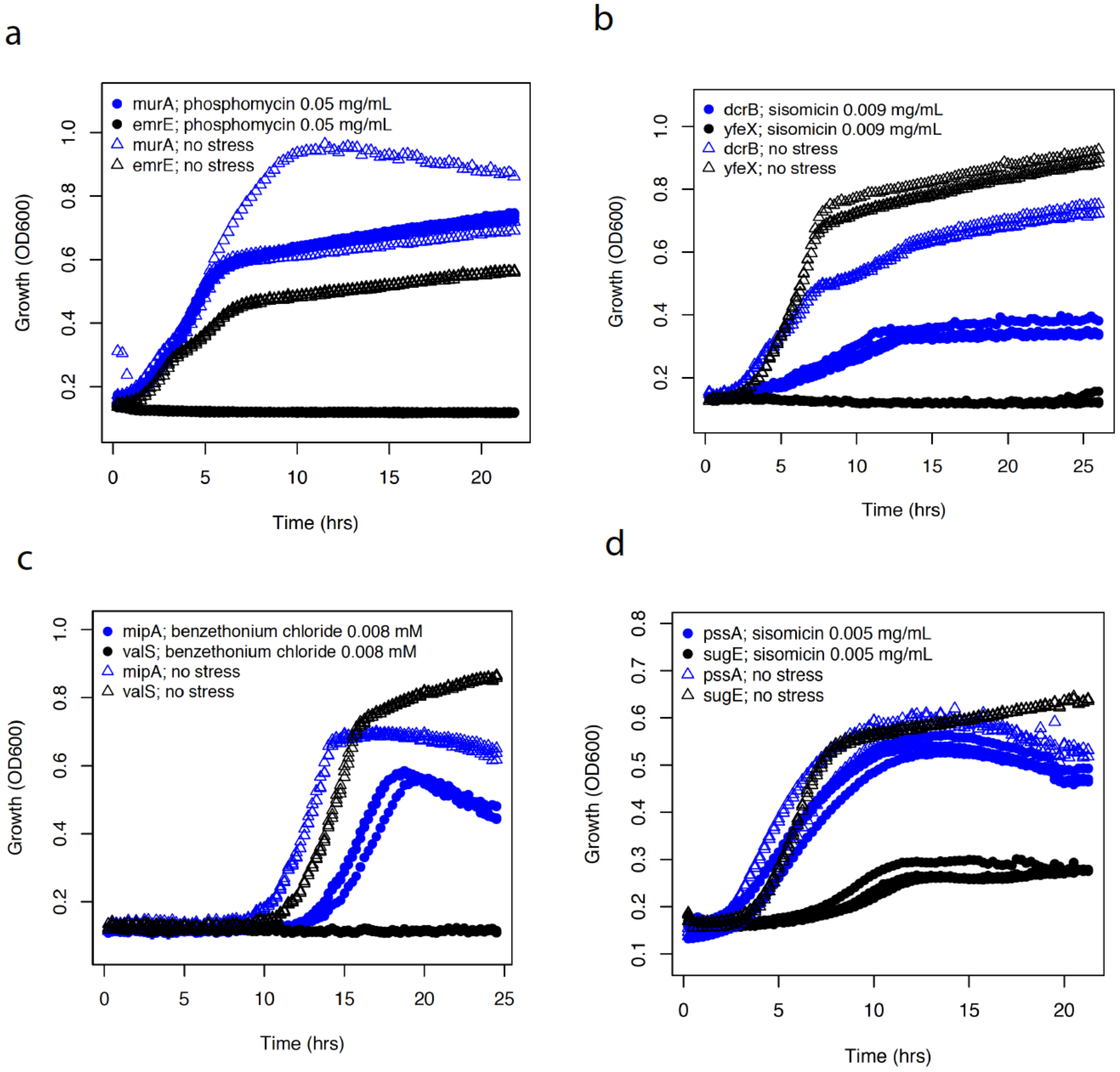

Supplementary Figure 5. Additional validation growth curves for Dub-seq high scoring genes. (a) Growth of *E. coli* overexpressing *murA* under phosphomycin stress; *emrE* is a control. (b) Growth of *E. coli* overexpressing *dcrB* under sisomicin stress; *yfeX* is a control. (c) Growth of *E. coli* overexpressing *mipA* under benzethonium chloride stress; *valS* is used as a control. (d) Growth of *E. coli* overexpressing *pssA* under sisomicin stress; *sugE* is used as a control. Source data are provided as a Source Data file.

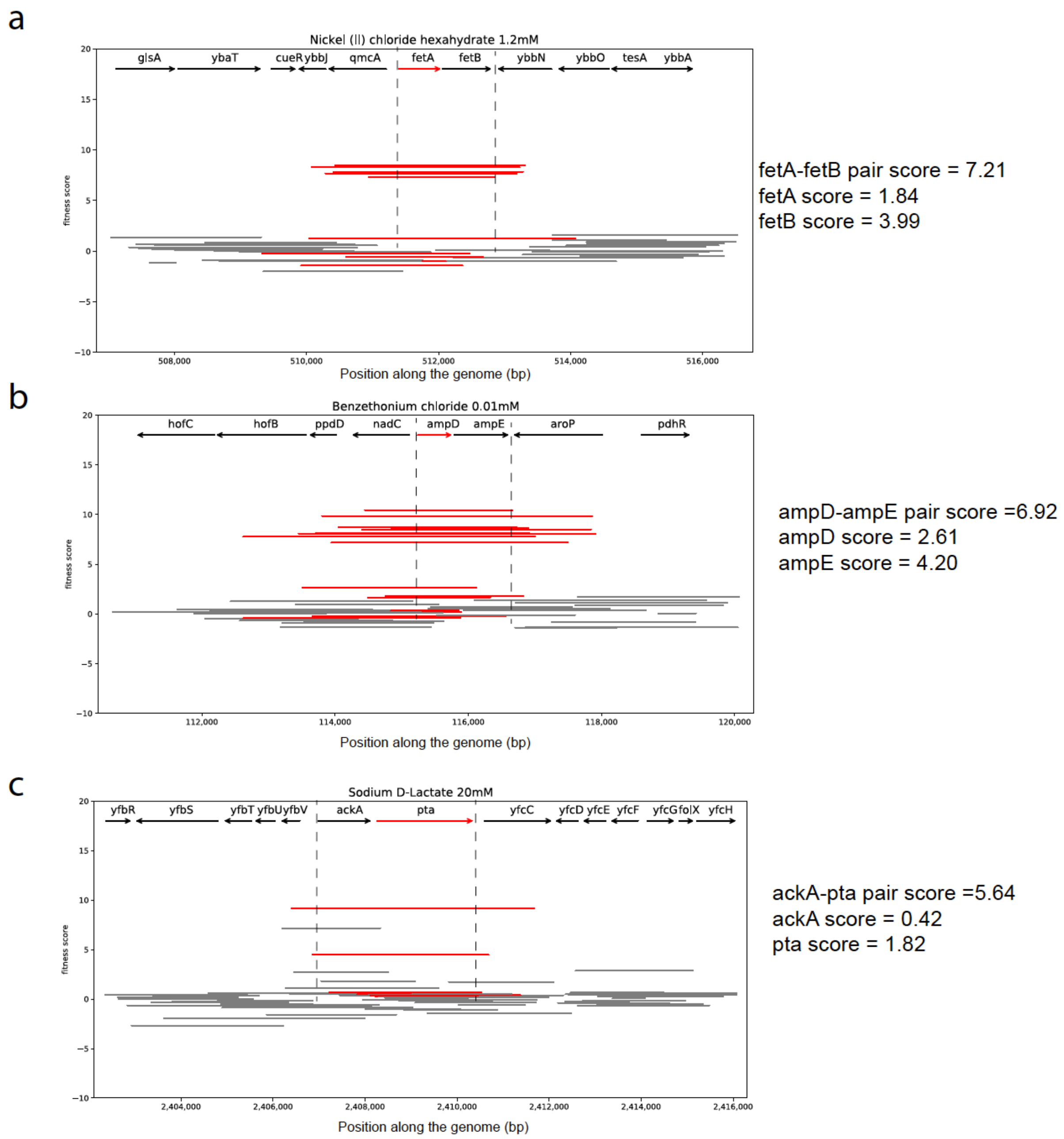

Supplementary Figure 6. Dub-seq gene-pair fitness scores: Dub-seq fragment (strain) data (y-axis) for region surrounding gene-pair of interest (x-axis). The covered fragments are shown in red and partially covered gene-pair-neighborhood fragments are shown in gray. The regression scores each gene-pair of interest are shown next to each plot. Compare scores for (a) *fetA and fetB with fetA-fetB pair* with (b) *ampD and ampE, with ampD-ampE pair* and (c) *ackA and pta* with ackA-pta pair. We looked for the scores for fragments containing more than one gene that are significantly greater than the inferred sum of score of the constituent genes.

a

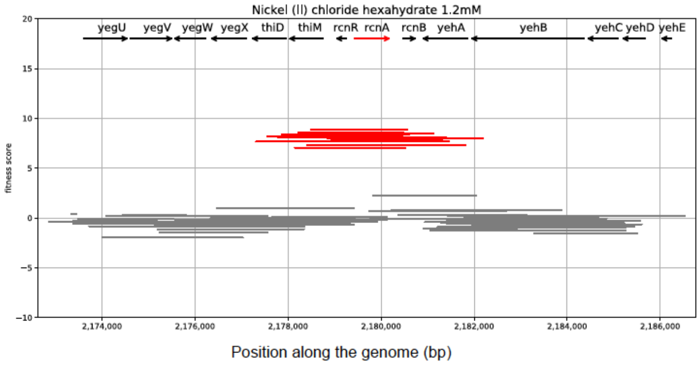

gene_name	rcnA
locus_tag	BW25113_2106
score_nnls	7.98483
score_ridge	5.87525
score_lasso	7.82251
score_enet	6.73892

b

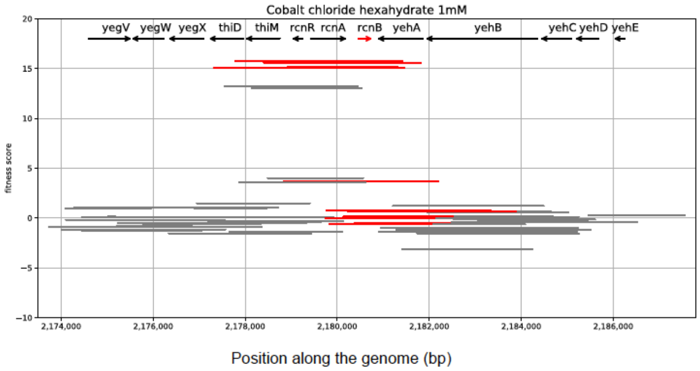

gene_name	rcnB
locus_tag	BW25113_2107
score_nnls	1.90233
score_ridge	5.68672
score_lasso	7.47208
score_enet	6.04639

c

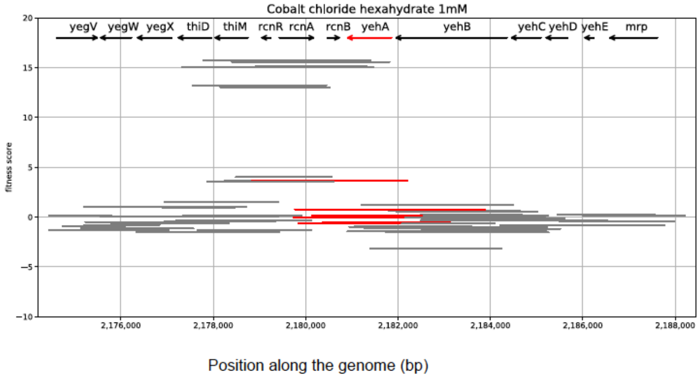

gene_name	yehA
locus_tag	BW25113_2108
score_nnls	0
score_ridge	-5.39295
score_lasso	-7.65625
score_enet	-5.86752

Supplementary Figure 7: Gene score estimation approaches: Example gene scores for (a) *rcnA* (b) *rcnB* and (c) *yehA* showing data over fitting and shrinkage by ridge, lasso and elastic net regularization methods. Left, Dub-seq viewer for fragments covering a specific gene completely (red), compared to partially covering or gene-neighborhood fragments (gray). The gene scores estimated using different methods are shown on right. The gene scores highlighted in blue lines indicate issues of regularization methods (see Supplementary Note 1).
