# Supplementary material for: Dual-barcoded shotgun expression library sequencing for high-throughput characterization of functional traits in bacteria
Source: Nat Commun. 2019 Jan 18;10:308. doi: 10.1038/s41467-018-08177-8 (PMC6338753; doi:10.1038/s41467-018-08177-8)
Supplement: Supplementary file 3 — Description of Additional Supplementary Files [file 41467_2018_8177_MOESM3_ESM.pdf]

Description of Additional Supplementary Files

File Name: Supplementary Data 1
Description: List of 135 genes not represented in E. coli Dubseq library

File Name: Supplementary Table 2
Description: List of protein coding genes with details on number of Dub-seq fragments covering the gene, and if the gene is essential (according to the Keio library, PMID: 16738554), has RB-TnSeq data (PMID: 29769716) and has Dub-seq data (this work)

File Name: Supplementary Data 3
Description: Filtered gene scores for reliable effects in Dub-seq dataset and if they have representative data in RB-TnSeq mutant library (PMID:29769716)

File Name: Supplementary Data 4
Description: List of genes whose high dosage is known to yield positive fitness effects

File Name: Supplementary Data 5
Description: Novel gene-function associations with fitness score >=4; hypothesis and general notes

File Name: Supplementary Data 6
Description: List of primers used in this work

File Name: Supplementary Data 7
Description: List of plasmids used in this work

File Name: Supplementary Data 8
Description: List of strains used in this work
