# Supplementary material for: Dual-barcoded shotgun expression library sequencing for high-throughput characterization of functional traits in bacteria
Source: Nat Commun. 2019 Jan 18;10:308. doi: 10.1038/s41467-018-08177-8 (PMC6338753; doi:10.1038/s41467-018-08177-8)
Supplement: Supplementary file 13 — Reporting Summary [file 41467_2018_8177_MOESM13_ESM.pdf]

nature research

Corresponding author(s): vkmutalik@lbl.gov
aparkin@lbl.gov

Reporting Summary

Nature Research wishes to improve the reproducibility of the work that we publish. This form provides structure for consistency and transparency in reporting. For further information on Nature Research policies, see Authors & Referees and the Editorial Policy Checklist.

Statistical parameters

When statistical analyses are reported, confirm that the following items are present in the relevant location (e.g. figure legend, table legend, main text, or Methods section).

n/a	Confirmed	
☐	☒	The exact sample size (*n*) for each experimental group/condition, given as a discrete number and unit of measurement
☐	☒	An indication of whether measurements were taken from distinct samples or whether the same sample was measured repeatedly
☐	☒	The statistical test(s) used AND whether they are one- or two-sided<br>*Only common tests should be described solely by name; describe more complex techniques in the Methods section.*
☒	☐	A description of all covariates tested
☐	☒	A description of any assumptions or corrections, such as tests of normality and adjustment for multiple comparisons
☒	☐	A full description of the statistics including central tendency (e.g. means) or other basic estimates (e.g. regression coefficient) AND variation (e.g. standard deviation) or associated estimates of uncertainty (e.g. confidence intervals)
☒	☐	For null hypothesis testing, the test statistic (e.g. *F*, *t*, *r*) with confidence intervals, effect sizes, degrees of freedom and *P* value noted<br>*Give P values as exact values whenever suitable.*
☒	☐	For Bayesian analysis, information on the choice of priors and Markov chain Monte Carlo settings
☒	☐	For hierarchical and complex designs, identification of the appropriate level for tests and full reporting of outcomes
☒	☐	Estimates of effect sizes (e.g. Cohen's *d*, Pearson's *r*), indicating how they were calculated
☒	☐	Clearly defined error bars<br>*State explicitly what error bars represent (e.g. SD, SE, CI)*

Our web collection on statistics for biologists may be useful.

Software and code

Policy information about availability of computer code

Data collection	Code for processing Dub-seq data is available at https://github.com/psnovichkov/DubSeq
Data analysis	Code for processing Dub-seq data is available at https://github.com/psnovichkov/DubSeq<br>Also, the R code to compute the high-confidence effects is in cons.R (in https://doi.org/10.6084/m9.figshare.6752753.v1). The noise computation relies on the counts, which are not included in the<br>R image. These files are in subdirectories of data/dubseq, i.e.<br>data/dubseq/FEBA_133/IT001.fscore.tsv

For manuscripts utilizing custom algorithms or software that are central to the research but not yet described in published literature, software must be made available to editors/reviewers upon request. We strongly encourage code deposition in a community repository (e.g. GitHub). See the Nature Research guidelines for submitting code & software for further information.

Data

Policy information about availability of data

All manuscripts must include a data availability statement. This statement should provide the following information, where applicable:

- Accession codes, unique identifiers, or web links for publicly available datasets
- A list of figures that have associated raw data
- A description of any restrictions on data availability

Complete data from all experiments (read counts per barcode, fragment scores and gene scores) is deposited here: [https://doi.org/10.6084/m9.figshare.6752753.v1]

The source data underlying Figs 2b–d, 3c-d, 4 and 5a-f and Supplementary Figs 1a-c, 2a-b, 3a-b, 5a-d and 6a-c are provided as a Source Data file.

Link to website with supplementary information and bulk data downloads: http://genomics.lbl.gov/supplemental/dubseq18/

Field-specific reporting

Please select the best fit for your research. If you are not sure, read the appropriate sections before making your selection.

☒ Life sciences ☐ Behavioural & social sciences ☐ Ecological, evolutionary & environmental sciences

For a reference copy of the document with all sections, see nature.com/authors/policies/ReportingSummary-flat.pdf

Life sciences study design

All studies must disclose on these points even when the disclosure is negative.

Sample size	Not applicable.
Data exclusions	None.
Replication	Some of the DubSeq assays had exact replicates. For mutant phenotypes in growth curves, all effects were confirmed on another day.
Randomization	Not applicable.
Blinding	Not relevant (data collection was automated).

Reporting for specific materials, systems and methods

Materials & experimental systems

n/a	Involved in the study	
☐	☒	Unique biological materials
☒	☐	Antibodies
☒	☐	Eukaryotic cell lines
☒	☐	Palaeontology
☒	☐	Animals and other organisms
☒	☐	Human research participants

Methods

n/a	Involved in the study	
☒	☐	ChIP-seq
☒	☐	Flow cytometry
☒	☐	MRI-based neuroimaging

Unique biological materials

Policy information about availability of materials

Obtaining unique materials	The E. coli Dub-seq library is available from the authors.
